# Musculoskeletal Screening of the Lumbopelvic Complex Among Male University-Level Fast Bowlers: A Cross-Sectional Study

**DOI:** 10.7759/cureus.85514

**Published:** 2025-06-07

**Authors:** Hari Kirthen, Geetha Sudha, Sai Aditya Raman, Arun Chelladurai, Sanketh Abba, Jibu George Varghese, Keddin Alwar Thiagarajan

**Affiliations:** 1 Sports and Exercise Sciences, Sri Ramachandra Institute of Higher Education and Research, Chennai, IND; 2 Arthroscopy and Sports Medicine, Sri Ramachandra Institute of Higher Education and Research, Chennai, IND

**Keywords:** biomechanical adaptations, cricket, fast bowling, injury prevention, lumbopelvic complex, range of motion, sacroiliac joint dysfunction

## Abstract

Background and aim: Fast bowling in cricket involves intense movements, stressing the lumbopelvic complex, crucial for force transfer, and the sacroiliac joint (SIJ), making it prone to dysfunction. Repetitive actions may alter the range of motion and SIJ function, risking injury. Assessing these structures is essential to detect adaptations and dysfunctions, enabling interventions to optimize performance and prevent injuries. This study aimed to assess lumbopelvic and hip range of motion and evaluate SIJ function in university-level fast bowlers and assess the injury risk with regard to these factors.

Methodology: Forty-four male university-level fast bowlers (aged 18-26 years) underwent hip and lumbar spine range of motion testing using a bubble inclinometer and goniometer. Standing and seated flexion tests were used to evaluate SIJ function. Descriptive statistics compared the range of motion (ROM) to normative values, while SIJ prevalence was evaluated with frequency distributions and 95% confidence intervals, and test agreement was assessed using concordance counts.

Results: Compared to normative values, fast bowlers showed higher lumbar flexion (58.86±7.28° vs. 51.5°), thoracolumbar rotation (59.16±5.70°-60.80±7.14° vs. 44.5°-44.9°), and hip extension (24.80±4.36°-25.05±3.48° vs. 19.4°). Lower ROM occurred in hip internal rotation (30.41±5.76°-29.48±5.11° vs. 36.2°) and left lateral flexion (22.77±4.27° vs. 25.6°). SIJ dysfunction prevalence was 22.7 (standing flexion test, 95% CI: 12.7, 36.8) and 15.9% (seated flexion test, 95% CI: 8.0, 29.5) with 70% test agreement.

Conclusion: University-level male fast bowlers exhibit sport-specific ROM adaptations and notable SIJ dysfunction prevalence, indicating potential injury risks. Routine SIJ screening and targeted rehabilitation, including dynamic stretching, core stabilization, and manual therapy, may enhance performance and reduce injuries.

## Introduction

Cricket fast bowlers, crucial for delivering high-velocity balls and exploiting pitch conditions to challenge batsmen, significantly influence match outcomes but face elevated risks of overuse injuries due to the biomechanical demands of their techniques [1]. Fast bowling actions, classified as front-on, side-on, semi-open, or mixed based on back foot contact, influence biomechanical patterns and trunk motion in fast bowlers, contributing to significant loading on the lumbopelvic region [1,2]. The lumbopelvic region plays a critical role in generating and transferring force from the lower body to the upper body during the bowling action, thereby sustaining substantial biomechanical loads [1]. Burnett et al. and Ferdinands et al. demonstrate that repetitive fast bowling movements generate unique stress patterns in the lumbopelvic complex, leading to ROM adaptations that may influence performance while increasing injury risk [3,4].

The sacroiliac joint (SIJ) contributes to 10-25% of lower back pain in athletes and sustains high stress as a critical link transferring forces between the lower extremities and spine during fast bowling [5,6]. SIJ dysfunction is an under-assessed issue in fast bowlers; therefore, it is vital to assess its function through reliable and valid tests, such as the standing and seated flexion tests, which demonstrate good construct validity and inter-examiner reliability [7,8]. However, SIJ dysfunction is an under-assessed issue in fast bowlers; therefore, it is vital to assess its function through tests such as the standing and seated flexion tests.

This study aimed to examine the ROM differences in the lumbopelvic complex and also assess the presence of SIJ dysfunction in Indian university-level male fast bowlers by using standing and seated flexion tests, in comparison to the available normative values. We hypothesized that fast bowlers would exhibit specific ROM adaptations due to repetitive, intense fast bowling action, display a higher prevalence of SIJ dysfunction, and face increased risk of injuries, including lower back pain, side strain, stress fractures, and sacroiliitis, due to lumbopelvic stress [2,5].

The findings from this study will be helpful for sports scientists, coaches, physiotherapists, and strength and conditioning specialists in the creation of specific training and rehabilitation programs, which can further help athletes to maintain functional ROM and improve joint stability, subsequently reducing the risk of injury. They can also be used to create evidence-based protocols for assessing and managing ROM and SIJ function. This study also lays the groundwork for future research to be done by establishing a benchmark for specific ROM and SIJ function, which helps encourage more in-depth investigations into the specific adaptations and biomechanics involved in fast bowling.

## Materials and methods

Study design

In this cross-sectional study, lumbopelvic and hip range of motion (ROM) and sacroiliac joint (SIJ) dysfunction were assessed at a single time point in male university-level fast bowlers. The study was performed at the Sri Ramachandra Institute of Higher Education and Research, Chennai, India. Data were collected between January and March 2025.

Sample size

Using a power calculation performed with SPSS software version 26 (Chicago, IL: IBM Corp.), and based on a mean hip flexion ROM of 71.3°±13.4° (non-bowling side) from Stuelcken et al., with 6% relative precision and a 95% confidence level, we estimated a required sample size of approximately 38, rounded to 40 for this study [9].

Inclusion and exclusion criteria

Active university-level male fast bowlers aged 18-26 years who had played cricket for more than two years and were currently practicing at least three times per week were included in this study. Individuals were excluded if they had a recent injury or surgery, were outside the specified age range, practiced less than three times per week, or had a history of bowling-related injuries to the lower back, pelvis, or hips within the past six months or surgeries related to these areas within the past two years.

Procedure

**Figure 1:**
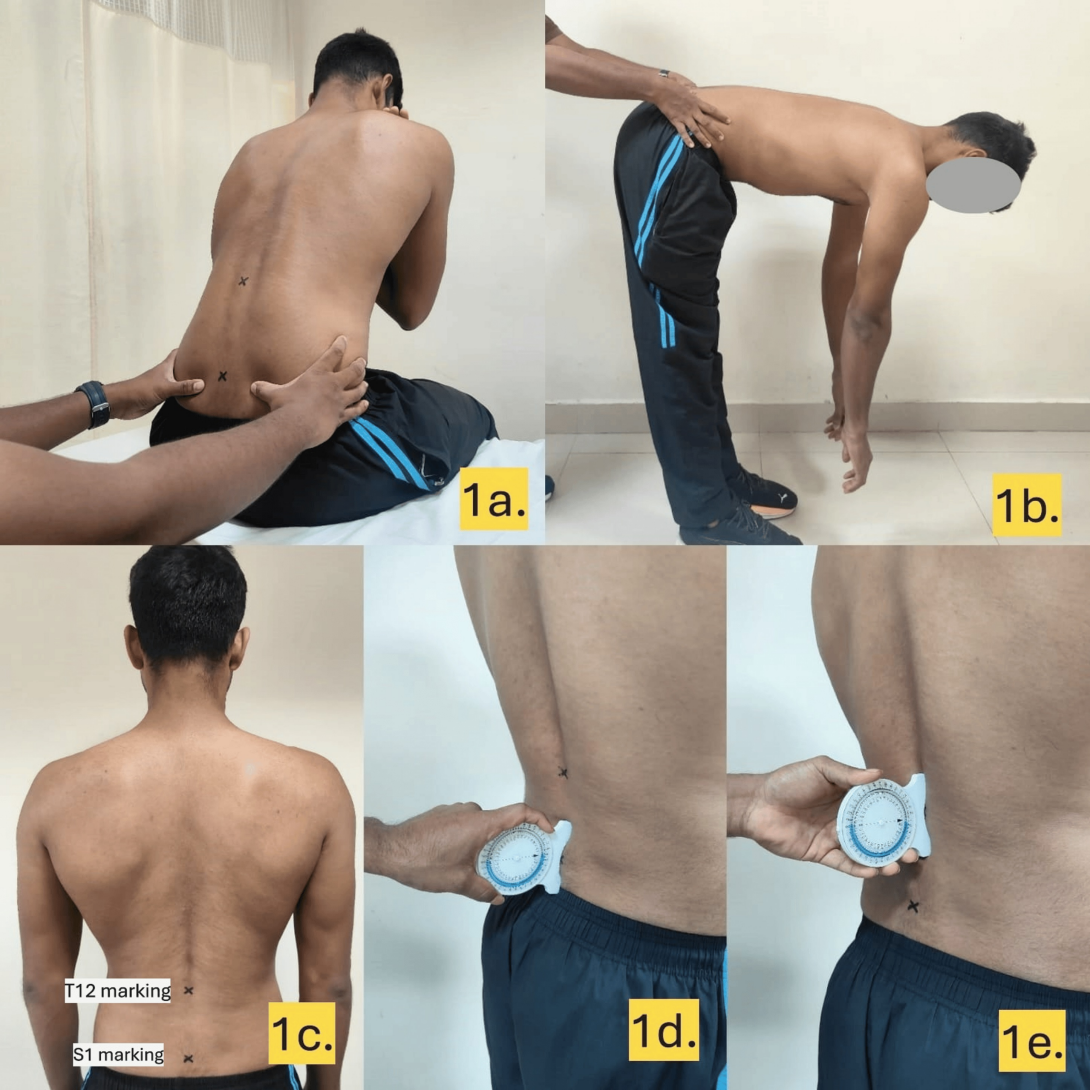
Tests and steps involved in the assessment. The images show (a) seated flexion test, (b) standing flexion test, (c) T12 and S1 marking, (d) bubble inclinometer placement at S1, and (e) bubble inclinometer placement at T12.

Male university-level fast bowlers were recruited and assessed from the Sri Ramachandra Institute of Higher Education and Research, Chennai, India, between January and March 2025. Lumbar ROM was measured using a bubble inclinometer at T12 and S1, calculating ROM by subtracting S1 from T12 angles. Participants performed maximal forward flexion, backward extension, and lateral flexion (standing) and thoracolumbar rotation (locked lumbar rotation test). Hip ROM was assessed with a goniometer, using the following landmarks: for flexion (supine, knee to chest) and extension (prone, leg lift), the fulcrum was placed at the greater trochanter, stationary arm along the pelvis midline, and moving arm along the femur midline; for adduction/abduction (supine, leg movement), the fulcrum was at the anterior superior iliac spine, stationary arm between both iliac spines, and moving arm along the femur toward the patella; and for internal/external rotation (seated, 90° knee flexion), the fulcrum was at the patella, stationary arm vertical, and moving arm along the tibia. SIJ dysfunction was evaluated by a physiotherapist using standing and seated flexion tests, assessing posterior superior iliac spine (PSIS) asymmetry during forward bending. Measurements were conducted by a sports physiotherapist and were blinded to the participants' bowling history to minimize bias. There was no missing data as all the participants completed all assessments. Figures 1a-1e highlight some of the tests and steps involved in the assessment.

Data analysis

Data were analyzed using IBM SPSS Statistics version 26. Lumbopelvic and hip range of motion (ROM) were summarized using means, standard deviations (SDs), and ranges for measures including flexion, extension, lateral flexion, and rotation. These descriptive statistics were compared with normative values from existing literature to contextualize findings, noting that referenced studies primarily involved non-Indian populations or non-athletic samples, limiting direct applicability [10-12]. Frequency distributions and 95% confidence intervals (CIs) were calculated to evaluate the prevalence of sacroiliac joint (SIJ) dysfunction, based on indicators such as asymmetry and pain provocation tests. The agreement between standing and seated flexion tests for SIJ dysfunction was assessed using concordance counts. No normality tests or inferential statistics were performed as this study focused on descriptive comparisons.

## Results

Participant flow

**Figure 2:**
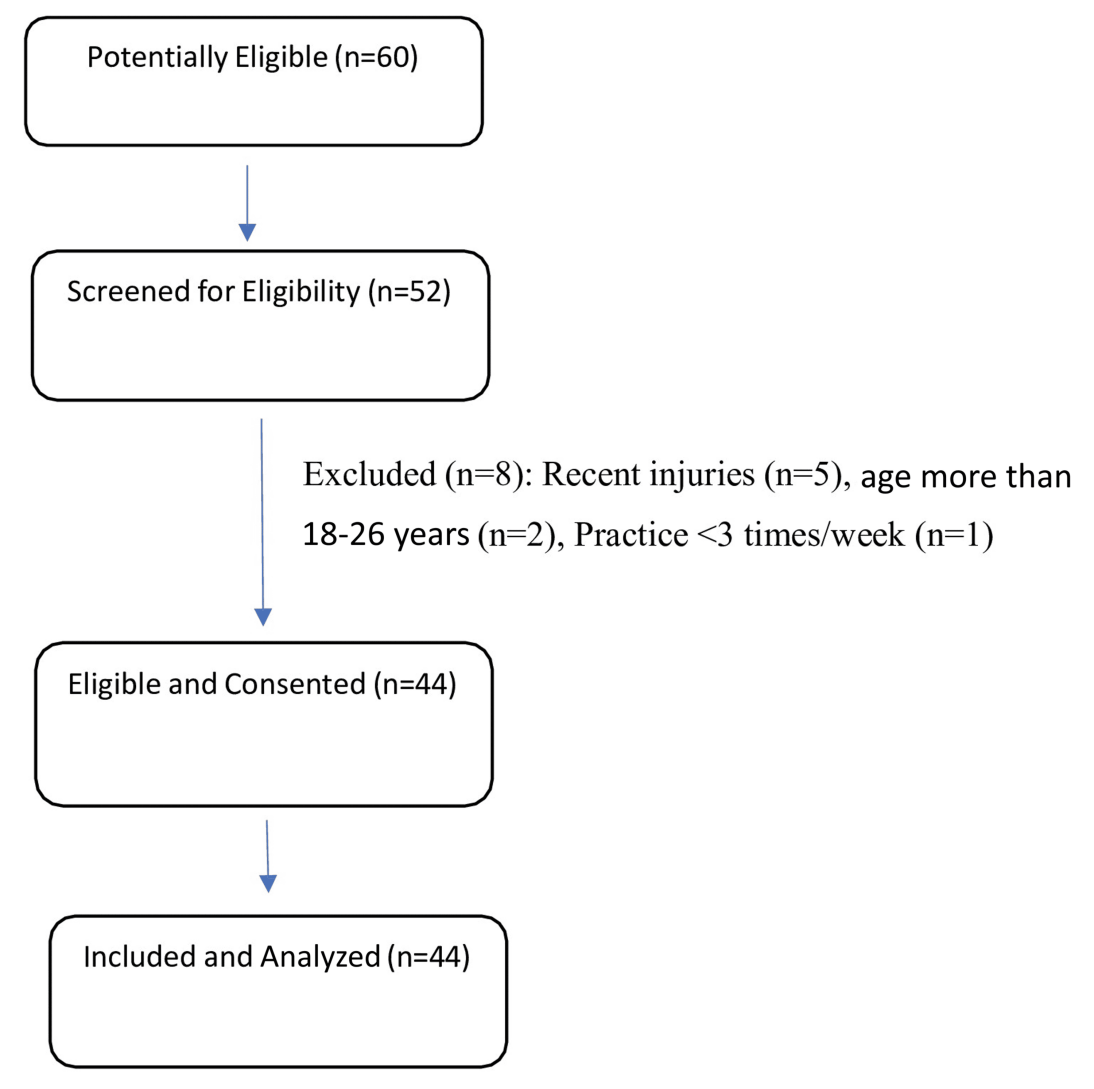
Flow diagram of participant selection for a cross-sectional study of university-level fast bowlers, showing numbers screened, included, and reasons for exclusion.

Of 60 university-level fast bowlers invited, 52 were screened for eligibility, and 44 met inclusion criteria and were included in the study. Exclusions were made due to recent injuries (n=5), age more than 18-26 years (n=2), or insufficient practice frequency (n=1). All 44 participants completed assessments and were analyzed as shown in Figure 2.

Demographic and descriptive statistics

Participants (n=44) were all male and right-handed fast bowlers with a mean age of 21.4±1.8 years and a mean bowling experience of 5.1±2.7 years (Table 1). Table 2 summarizes ROM for 44 fast bowlers. All 44 participants completed lumbar and hip ROM assessments and SIJ dysfunction tests, with no missing data for any variable of interest (Table 2).

**Table 1:** Demographic characteristics of the participants.

Characteristics	Values
Age in years (mean±SD)	21.4±1.8
Age range	18-24
Years of bowling (mean±SD)	5.1±2.7
Years of bowling range	2-11
Gender (male), n (%)	44 (100%)
Bowling side (right), n (%)	44 (100%)

**Table 2:** Descriptive statistics of the participants.

Variables	n	Mean (°)	SD (°)	Range (°)
Lumbar spine				
Flexion	44	58.86	7.28	45-72
Extension	44	27.91	4.40	21-37
Thoracolumbar rotation left	44	59.16	5.70	45-69
Thoracolumbar rotation right	44	60.80	7.14	43-73
Lateral flexion left	44	22.77	4.27	15-34
Lateral flexion right	44	26.20	4.10	17-34
Hip				
Flexion left	44	122.39	8.62	103-137
Flexion right	44	123.57	7.24	107-137
Extension left	44	24.80	4.36	17-34
Extension right	44	25.05	3.48	18-31
Internal rotation left	44	30.41	5.76	21-41
Internal rotation right	44	29.48	5.11	21-39
External rotation left	44	44.39	7.58	30-58
External rotation right	44	43.91	7.44	29-56
Adduction left	44	25.36	3.72	20-33
Adduction right	44	26.18	3.69	19-35
Abduction left	44	39.52	4.90	28-48
Abduction right	44	41.14	4.71	31-49

Comparison to normative values

In Table 3, the ROM measurements are compared to normative values from literature [10-12]. Lumbar flexion (58.86±7.28° vs. 51.5°) when compared to the normative value was higher, whereas lumbar extension compared to one value was higher (27.91±4.40° vs. 23.0°), but not with respect to another normative value (31.9°). Thoracolumbar rotation was notably higher in the left (left: 59.16±5.70° vs. 44.9°; right: 60.80±7.14° vs. 44.5°). While right lateral flexion (26.20±4.10° vs. 26.6°) was similar, left lateral flexion was lower (22.77±4.27° vs. 25.6°).

**Table 3:** Descriptive comparison of ROM to normative values.

Variables	Normative value (°)	Mean±SD (°)
Lumbar spine		
Flexion	51.5	58.86±7.28
Extension	23.0	27.91±4.40
Extension	31.9	27.91±4.40
Thoracolumbar rotation left	44.9	59.16±5.70
Thoracolumbar rotation right	44.5	60.80±7.14
Lateral flexion left	25.6	22.77±4.27
Lateral flexion right	26.6	26.20±4.10
Hip		
Flexion left	123.7	122.39±8.62
Flexion right	123.7	123.57±7.24
Extension left	19.4	24.80±4.36
Extension right	19.4	25.05±3.48
Internal rotation left	36.2	30.41±5.76
Internal rotation right	36.2	29.48±5.11
External rotation left	41.2	44.39±7.58
External rotation right	41.2	43.91±7.44
Adduction left	28.1	25.36±3.72
Adduction right	28.1	26.18±3.69
Abduction left	41.8	39.52±4.90
Abduction right	41.8	41.14±4.71

Hip flexion was within normative (left: 122.39±8.62° vs. 123.7°; right: 123.57±7.24° vs. 123.7°). Hip extension was higher (left: 24.80±4.36° vs. 19.4°; right: 25.05±3.48° vs. 19.4°). There was lower hip internal rotation (left: 30.41±5.76° vs. 36.2°; right: 29.48±5.11° vs. 36.2°) and higher hip external rotation (left: 44.39±7.58° vs. 41.2°; right: 43.91±7.44° vs. 41.2°). Left abduction (39.52±4.90° vs. 41.8°) and hip adduction (left: 25.36±3.72° vs. 28.1°; right: 26.18±3.69° vs. 28.1°) were both lower, but right abduction was subsequently similar (41.14±4.71° vs. 41.8°).

**Figure 3:**
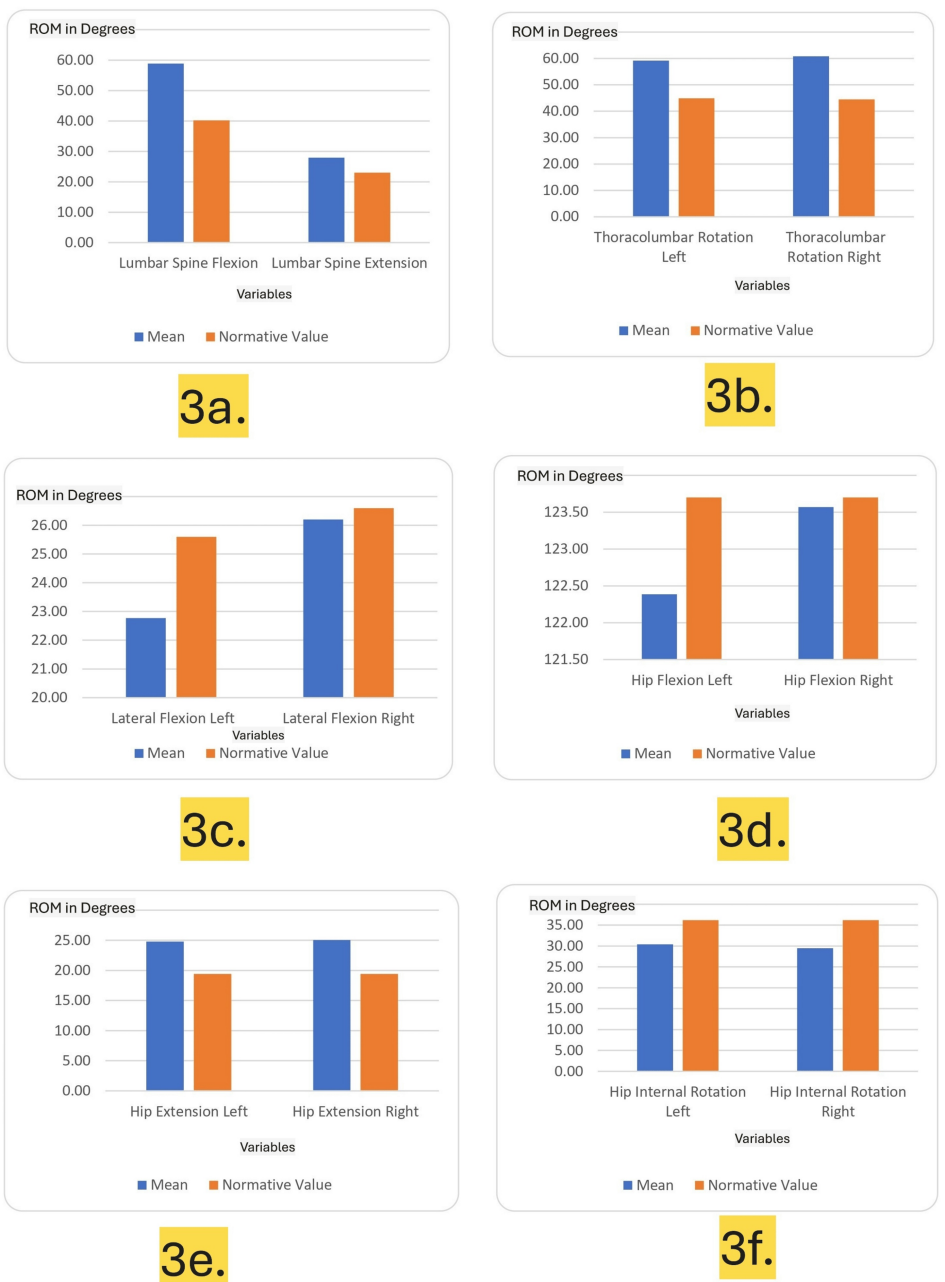
Lumbopelvic and hip range of motion (ROM) measurements in university-level fast bowlers compared to normative values. The images show (a) lumbar spine flexion and extension, (b) thoracolumbar rotation (left and right), (c) lateral flexion (left and right), (d) hip flexion (left and right), (e) hip extension (left and right), and (f) hip internal rotation (left and right). The x-axis shows the variables taken into consideration and the y-axis shows the range of motion (ROM) in degrees achieved.

**Figure 4:**
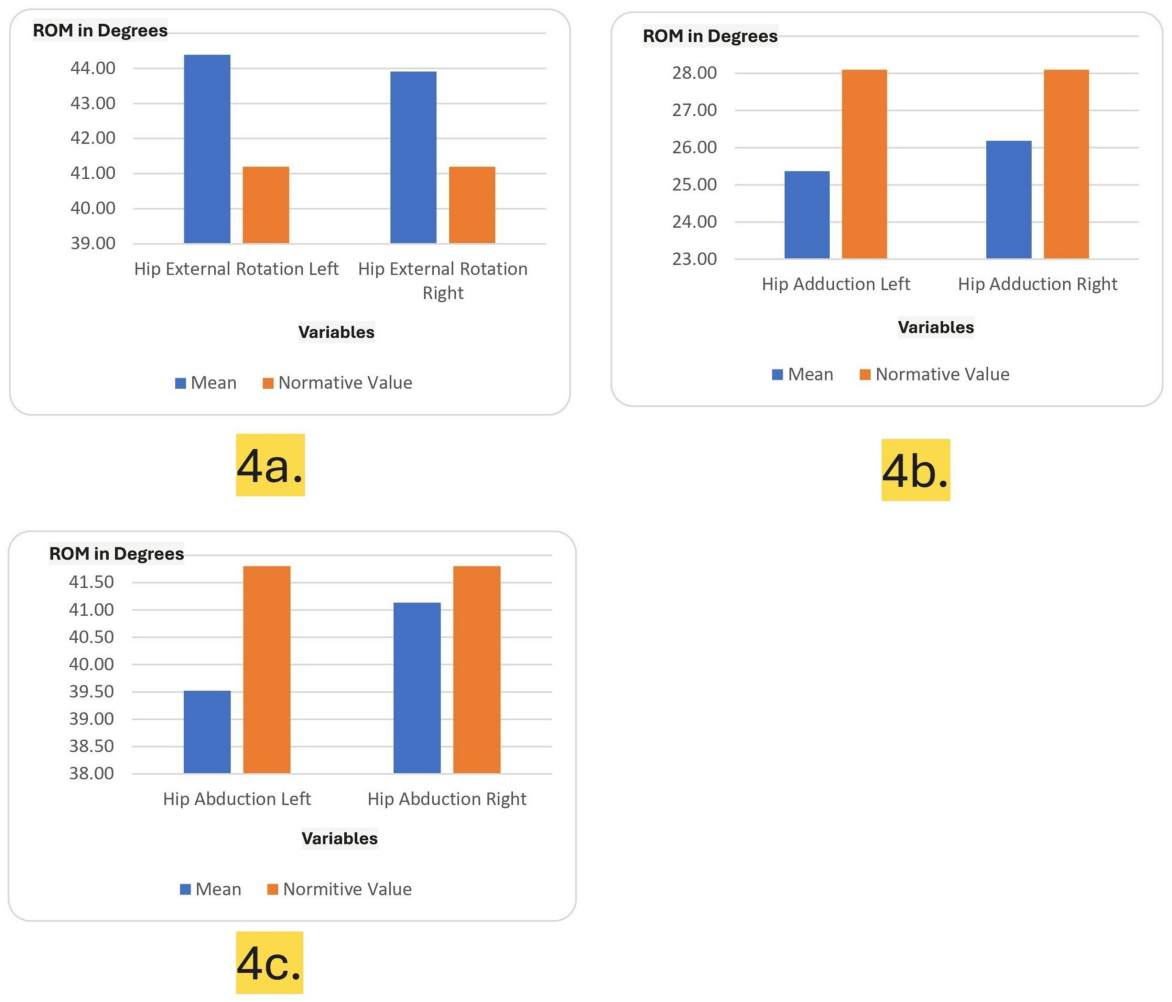
Hip range of motion (ROM) measurements in university-level fast bowlers compared to normative values. The x-axis shows the variables taken into consideration and the y-axis shows the range of motion (ROM) in degrees achieved. The images show (a) hip external rotation (left and right), (b) hip adduction (left and right), and (c) hip abduction (left and right).

Figures 3a-3f and Figures 4a-4c illustrate the range of motion (ROM) measurements for the lumbar spine and hip in university-level fast bowlers, comparing mean values to normative standards across multiple variables such as flexion, extension, rotation, adduction, and abduction.

Sacroiliac joint dysfunction assessment

The standing flexion test identified SIJ dysfunction in 10 participants (22.7%, 95% CI: 12.7, 36.8), with three (6.8%) positive to the left and seven (15.9%) positive to the right. The seated flexion test detected dysfunction in seven participants (15.9%, 95% CI: 8.0, 29.5), with two (4.5%) positive to the left and five (11.4%) positive to the right (Table 4). Most participants (34, 77.3%) tested negative for SIJ dysfunction in both tests, indicating no dysfunction. Among the 10 participants positive on the standing test, seven (70%) were also positive on the seated test, while three (30%) were negative on the seated test (Table 5). No participants were positive on the seated test alone.

**Table 4:** Frequency of sacroiliac joint dysfunction, positive to left/right indicates SIJ dysfunction to the respective side.

Test	Outcome	Frequency	Percentage	95% CI
Standing flexion	Negative	34	77.3	63.2-87.3
	Positive to left	3	6.8	2.3-18.3
	Positive to right	7	15.9	8.0-29.5
Seated flexion	Negative	37	84.1	70.5-92.0
	Positive to left	2	4.5	1.2-15.0
	Positive to right	5	11.4	4.9-24.1

**Table 5:** Concordance of standing and seated flexion tests.

Test	Outcome	Frequency	Percentage	95% CI
Standing flexion	Negative	34	77.3	63.2-87.3
	Positive to left	3	6.8	2.3-18.3
	Positive to right	7	15.9	8.0-29.5
Seated flexion	Negative	37	84.1	70.5-92.0
	Positive to left	2	4.5	1.2-15.0
	Positive to right	5	11.4	4.9-24.1

## Discussion

The purpose of this study was to assess lumbopelvic range of motion (ROM) and sacroiliac joint (SIJ) function among male university-level fast bowlers. The findings highlight biomechanical adaptations in male fast bowlers due to the unique stresses of bowling action, suggesting potential injury risks based on the literature [5,6]. These descriptive results underscore the value of targeted screening and rehabilitation strategies, though the cross-sectional design limits causal inferences about injury risk.

Lumbar flexion (58.86±7.28° vs. 51.5°) was higher than normative values, possibly indicating increased forward mobility as a result of repetitive bowling mechanics. Lumbar extension was also seen to be greater than one normative value (27.91±4.40° vs. 23.0°) but lower than another (31.9°), suggesting normative benchmarks had variability and the need for sport-specific comparisons [10]. Lateral flexion to the left was lower (22.77±4.27° vs. 25.6°) while lateral flexion to the right was almost similar to the normative value (26.20±4.10° vs. 26.6°), possibly due to side dominance or restricted mobility. Enhanced thoracolumbar rotational mobility (left: 59.16±5.70° vs. 44.9°; right: 60.80±7.14° vs. 44.5°) was also present, which may be a result of the repetitive bowling mechanics. Force transfer is possible through these adaptations, which are critical for momentum in the delivery stride [13,14].

The mechanics of fast bowling, particularly mixed action types, influence ROM adaptations, such as increased thoracolumbar rotation and lumbar flexion [15,16]. Bayne et al. noted that adolescent fast bowlers experience significant lumbar loads during front-foot contact, contributing to enhanced lumbar ROM that may improve performance but suggests potential injury risks [17]. These descriptive findings highlight sport-specific adaptations, but the cross-sectional design precludes causal links to injury.

Hip flexion showed no significant deviation from normative values, but hip extension was higher bilaterally (left: 24.80±4.36° vs. 19.4°; right: 25.05±3.48° vs. 19.4°), indicating greater posterior chain mobility. Hip internal rotation was reduced (left: 30.41±5.76° vs. 36.2°; right: 29.48±5.11° vs. 36.2°), while external rotation was elevated (left: 44.39±7.58° vs. 41.2°; right: 43.91±7.44° vs. 41.2°). Hip adduction (left: 25.36±3.72° vs. 28.1°; right: 26.18±3.69° vs. 28.1°) and left abduction (39.52±4.90° vs. 41.8°) were also reduced. These adaptations, likely due to repetitive rotational forces in bowling, may be associated with increased lumbar stress, as restricted hip rotation can lead to compensatory lumbar movements, per the literature [18]. No statistical associations were tested due to the small sample size (n=44) and cross-sectional design.

Repetitive unilateral loading during fast bowling, particularly at front-foot contact, contributes to ROM asymmetries, such as reduced left lumbar lateral flexion (22.77±4.27° vs. 25.6°) [19]. These asymmetries may lead to uneven force distribution in the lumbopelvic complex, potentially increasing stress, as noted by Forrest et al. [20]. They can also disrupt force distribution in the lumbopelvic complex, predisposing to overuse injuries [21]. Altered lumbopelvic mechanics, including restricted hip internal rotation, may further elevate lumbar stress when core stability is inadequate [22,23]. These descriptive findings suggest the need for targeted interventions, but causal relationships with overuse injuries were not established due to the study’s design. These findings point out the importance of targeted interventions, such as dynamic stretching and core stabilization, to correct the deficits and improve the lumbopelvic function in fast bowlers [24,25].

Standing and seated flexion tests identified SIJ dysfunction prevalence (22.7% standing, 15.9% seated), consistent with athletic populations (10-25%) [5,6]. Right-sided dysfunction (15.9% standing, 11.4% seated) may reflect asymmetrical loading during bowling [19,26]. Our study’s 70% agreement between standing and seated flexion tests suggests that combining these tests, which assess SIJ mobility under different conditions, enhances screening reliability for fast bowlers, consistent with principles of SIJ diagnostic testing [6]. These descriptive findings highlight SIJ dysfunction in fast bowlers, potentially linked to reduced hip internal rotation (30.41±5.76°-29.48±5.11° vs. 36.2°), but no statistical associations with injury risk were tested due to the small sample and cross-sectional design.

In regard to the current rehabilitation protocols, stretching, active hip rotation drills, and leg raises have been demonstrated to be effective in reducing compensatory lumbar stress for the reduction of hip internal rotation and adduction deficits [25,26]. Ball et al. suggest a progressive loading program targeting lumbar spine injuries, which includes a bird dog variation to strengthen the core and stabilize the torso relative to the pelvis, thereby reducing excessive movement during bowling [26]. Neuromuscular exercises such as single-leg balance drills can provide neuromuscular training and will improve motor control, decreasing the risk of hip and groin injury known to be strongly related to SIJ dysfunction [25].

The reported manual therapy techniques to restore the joint's intended alignment, with reducing the proxy pain associated with SIJ dysfunction, include SIJ mobilizations and manipulation [6]. Mulligan concept movement with mobilization has shown promising results in reducing pain and stiffness during resting positions and weight-bearing functional activities [27]. Ball et al. usually conduct the strengthening of the device's gluteal, pelvic floor, and deep core muscle (e.g., transversus abdominis) for the development of better joint stability and load distribution [26]. In functional exercise, the bowling movement can be integrated with SIJ stability, such as resistance to hip external rotation, and lumbopelvic stabilization drills [20]. Additionally, the technique modification program is designed specifically for bowlers with a mixed action type. It aimed to reduce excessive trunk rotation and lumbar stress through biomechanical retraining, incorporating real-time feedback [16]. Dynamic stretching, rather than static stretching, accompanied by sport-specific warmups, is associated with increased ROM and neuromuscular facilitation without negative effects on power and speed in the fast bowling population [22]. This study helps in the characterization of lumbopelvic ROM and SIJ dysfunction in a unique cohort of Indian male university-level fast bowlers, providing valuable baseline data in the absence of normative values for this population.

Clinical implications

The results of this study have very important implications for sports scientists, physiotherapists, and coaches of elite fast bowlers. Lumbar and thoracic ROM increased, but training programs should emphasize maintaining these findings and compensatory injured areas of hip internal rotation, abduction, and left lumbar lateral flexion and abduction to prevent compensatory injuries [28]. Pre-season and in-season SIJ screening with both standing and seated flexion tests should be carried out to identify "at-risk" bowlers early. As bowlers’ action type and biomechanical profile vary, rehabilitation programs should be individualized, including mobility, stability, and correction of technique.

Limitations

This cross-sectional study cannot establish causality between lumbopelvic range of motion (ROM) adaptations, sacroiliac joint (SIJ) dysfunction, and injury risk [22]. The small sample size (n=44), determined to achieve descriptive precision, and the homogeneous sample (all male, right-handed university-level fast bowlers) limited the feasibility of inferential statistical analyses, such as testing associations or subgroup comparisons [9]. The absence of normative ROM and SIJ function data for Indian athletes further constrained the study to descriptive statistics, as comparisons with existing literature were limited by differences in population (e.g., non-Indian or non-athletic samples) [10-12]. Single-institution recruitment at Sri Ramachandra Institute of Higher Education and Research (SRIHER) may restrict generalizability, and the lack of assessor blinding introduces potential bias.

Future directions

The limitations of this study highlight the exploratory nature of the findings and underscore the need for future studies, such as randomized controlled trials with larger, more diverse samples, to investigate causal relationships and validate these observations. In regard to ROM deficits, SIJ dysfunction, and injuries in fast bowlers, longitudinal studies should explore causality [22]. Research pertaining to bowling action types (front on, side on, mixed) can optimize training [2,17]. 3D motion capture, for example, can help explain the load distribution [3,28]. A rehabilitation program that tests hip mobility and core stabilization might reduce lumbar stress [20,26]. Additionally, SIJ screening using standardized protocols could enhance both safety and athletic performance by identifying dysfunctions that may contribute to lower back issues [8,24,29]. Future RCTs with larger, diverse samples could explore causal relationships and injury risk.

## Conclusions

Male fast bowlers exhibited increased lumbar flexion (58.86° vs. 51.5°), thoracolumbar rotation (59.16°-60.80° vs. 44.5°-44.9°), hip extension (24.80°-25.05° vs. 19.4°), and 22.7% SIJ dysfunction prevalence, with reduced hip internal rotation (30.41°-29.48° vs. 36.2°), suggesting potential injury risks based on literature. These descriptive findings support routine screening, but the cross-sectional design, small sample (n=44), and limited Indian normative data prevent causal inferences. Future RCTs are needed to explore ROM/SIJ-injury risk relationships.
